# Supplementary material for: Disentangling metabolic functions of bacteria in the honey bee gut
Source: PLoS Biol. 2017 Dec 12;15(12):e2003467. doi: 10.1371/journal.pbio.2003467 (PMC5726620; doi:10.1371/journal.pbio.2003467)
Supplement: S8 Data — (ZIP) [file pbio.2003467.s008.zip › Z-score_plots/902 microbial substrate alpha-L-Rhamnopyranosyl-(1-2)-beta-D-galactopyranosyl-(1-2)-beta-D-glucur....pdf]

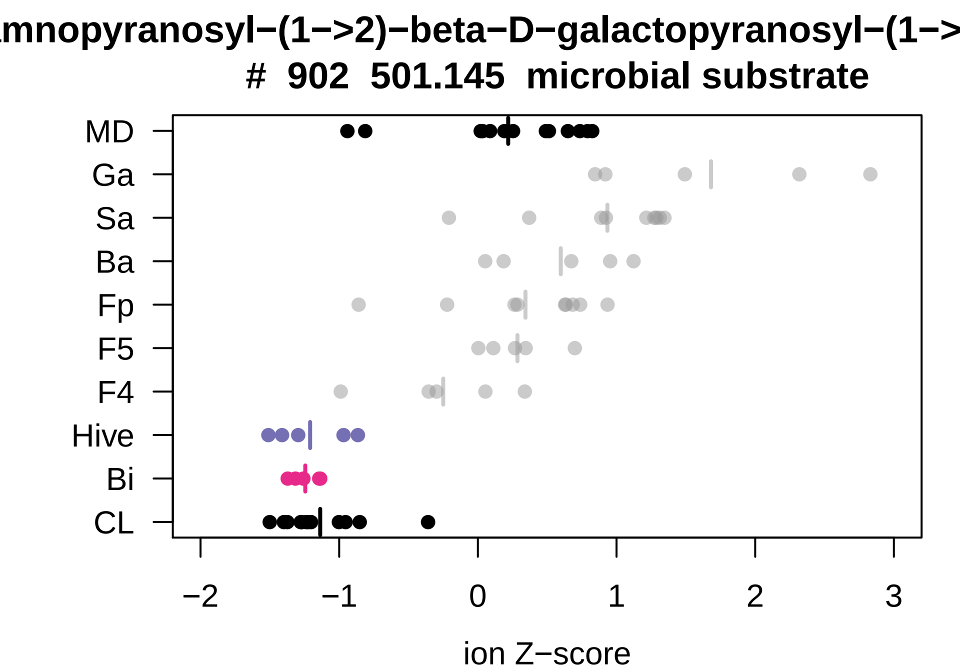
mnopyranosyl−(1−>2)−beta−D−galactopyranosyl−(1−>
902 501.145 microbial substrate
MD
Ga
Sa
Ba
Fp
F5
F4
Hive
Bi
CL
−2
−1
0
1
2
3
ion Z−score
